# Clinical analysis and long-term treatment monitoring of 3 patients with glycogen storage disease type Ib

**DOI:** 10.1186/s12920-021-00936-9

**Published:** 2021-03-17

**Authors:** Caiqi Du, Zhuoguang Li, Hong Wei, Min Zhang, Minghui Hu, Cai Zhang, Xiaoping Luo, Yan Liang

**Affiliations:** Department of Pediatrics, Tongji Hospital, Tongji Medical College, Huazhong University of Science and Technology, Wuhan, 430030 China

**Keywords:** Glycogen storage disease type Ib, *SLC37A4*, Genetic analysis, Follow-up, Treatment

## Abstract

**Background:**

To investigate the clinical and genetic characteristics of patients with glycogen storage disease type Ib (GSD Ib).

**Case presentation:**

This report retrospectively analyzed the clinical data of 3 patients with GSD Ib admitted into our hospital, and summarized their onset characteristics, clinical manifestations, related examinations and treatment as well as mutational spectrum. After gene sequencing, the diagnosis of GSD Ib was confirmed in all 3 patients. Five variants of *SLC37A4* gene were detected, of which c. 572C > T was the common variant and c. 680G > A was a novel variant. The 3 cases of GSD Ib were mainly affected by liver enlargement, growth retardation, etc., and all had a history of repeated infections. At the onset, patients mainly manifested as mildly elevated alanine-aminotransferase (ALT), accompanied by decreased absolute neutrophil count (ANC), hypertriglyceridemia, and metabolic disorders (hypoglycemia, hyperlactic acidemia, metabolic acidosis, etc.). After long-term treatment by oral uncooked cornstarch, the abnormal liver enzymes gradually returned to normal, and metabolic abnormalities were basically controlled most of the time. With increasing age, ANC of 2 patients decreased progressively, whereas the times of infections was reduced.

**Conclusions:**

We reported 3 cases with GSD Ib and a novel *SLC37A4* variant. The possibility of GSD type Ib should be kept on alert when a patient suffers recurrent infections, accompanied by hepatomegaly, elevated liver enzymes, hypoglycemia, dyslipidemia, and metabolic disorders.

## Background

Glycogen storage disease type I (GSDI) is a group of autosomal recessive inherited metabolic disorders with varying clinical severity caused by variants in the *G6PC* gene (OMIM #613742) or *SLC37A4* gene (OMIM # 602671), and the incidence is about 1:100,000 [1]. The *G6PC* gene variant causes the deficiency of glucose-6-phosphatase alpha (G6Pase-α) activity, which leads to GSD type Ia (OMIM#232200), accounting for about 80% of GSDI patients, whereas the *SLC37A4* gene variant causes the deficiency of glucose-6-phosphate transporter protein (G6PT), which underlies GSD type Ib (OMIM# 232220), accounting for about 20% of GSDI cases [1, 2].

Both type Ia and Ib are characterized by hepatomegaly and metabolic abnormalities such as hypoglycemia, hyperlipemia, lactic acidosis, and hyperuricemia. The *SLC37A4* gene is highly expressed in hematopoietic progenitor cells, its defect has a significant effect on myeloid progenitor cells [3]. At the same time, G6PT plays a role in the neutrophil homeostasis and function, endogenous glucose production is critical for neutrophil homeostasis, so the deficiency of G6PT can cause neutrophil apoptosis and neutropenia [4]. Therefore, GSD Ib patients manifest neutropenia and neutrophil dysfunction, and are prone to frequent infectious diseases, such as recurrent upper respiratory tract infections, oral and intestinal mucosal ulcers, and inflammatory bowel disease (IBD), etc. Therefore, different treatment plans are needed for GSD Ib patients [2, 5].

We report 3 additional cases of GSD Ib with *SLC37A4* variant, one of which had novel variant.

## Case presentation

The clinical features of the 3 patients were summarized in Tables 1, 2, and 3.

**Table 1:** Clinical baseline data and genetic features of the 3 patients with GSD type Ib

	Patient 1	Patient 2	Patient 3
Clinical baseline data			
Gender	Female	Male	Female
Age of onset (months)	1	8	30
Age at clinical diagnosis (months)	15	42	36
Course (months)	14	34	6
Age at genetic diagnosis (years)	12	13	11
Chief complaint	Abdominal distension	Upper respiratory tract infection, hepatomegaly	Short stature, hepatomegaly
Liver biopsy	PAS ( +)	PAS ( +)	PAS ( +)
Epinephrine tolerance test	Positive	Positive	Positive
Genotypes			
Base change	c.[1016G > A];[572C > T]	c.[572C > T]; [343G > A]	c.[870 + 5G > A];[680G > A]
Amino change	p.[G339D];[P191L]	p.[P191L];[G115R]	splicing; W227*
Exon	10;6	6;10	7;7
Novel	− / −	− / −	− /Our first report

**Table 2:** Comparison of clinical and biochemical indexes of 3 patients with GSD type Ib before and after treatment

	Patient 1			Patient 2			Patient 3		
	Clinical diagnosis	Genetic diagnosis	Last visit	Clinical diagnosis	Genetic diagnosis	Last visit	Clinical diagnosis	Genetic diagnosis	Last visit
Age (years)	1.25	12.33	15	3.5	13	14	3	10.5	11.75
Height (cm)	72	141.8	150	89	136	141.6	90	137.7	148.9
Height (SDS)	− 2.26	− 2.05	− 1.79	− 2.97	− 3.02	− 3.38	− 1.48	− 0.86	− 0.11
Growth velocity (cm/year)	–	6.30	3.07	–	4.95	5.6	–	6.36	8.96
Weight (Kg)	9	47.5	57.5	13	28.5	34	15	33.1	50
Weight (P)	10–25	50–75	75–90	10–25	< 3	< 3	50–75	25–50	90–97
BMI(kg/m2)	18.9	23.6	25.6	16.4	15.4	17.0	18.5	17.5	22.6
Sexual development									
Stages of breast development	B1	B3	B4	–	–	–	B1	B2	B3
Testicular volume (ml)	–	–	–	1	3	6	–	–	–
Bone age (years)	–	–	–	–	–	13	–	–	–
Complete blood count									
ANC (× 10^9^/L)	1.07	0.94	0.28	0.95	0.46	0.3	0.82	0.41	0.22
Liver function									
ALT (U/L)	75	10	25	50	37	30	37	28	19
AST (U/L)	103	12	17	46	28	20	54	30	18
Blood lipids									
TG (mmol/L)	8.84	3.12	2.03	5.53	3.56	2.1	3.92	4.89	2.91
HDL (mmol/L)	1.13	0.83	0.73	0.91	0.75	0.87	1.28	0.86	1.01
Glucose metabolism parameters									
Fasting glucose (mmol/L)	3.7	2.79	3.1	3.8	2.49	4.63	3.48	3.64	6.65
Lactic acid (mmol/L)	8.78	6.42	6.89	10.75	11.41	3.98	9.61	4.09	4.05
Pyruvic acid (umol/L)	52.8	269.1	263.4	284.7	509.3	135.2	297.4	297.4	168.1
Uric acid (μmol/L)	281.9	674	419	499	537	366	519	522	278
Blood gases									
PH	7.413	7.376	7.363	7.312	7.312	7.346	7.38	7.38	7.341
BE	− 8.5	− 5.8	− 6.4	− 10.9	− 10.9	− 1.1	− 1.1	− 1.1	− 2.8
Echocardiography									
Liver size (cm)	4	8.8	2	5	8	4.5	9	8	7
Kidney	Normal	Normal	Normal	Normal	Normal	Normal	Normal	Normal	Normal

Abbreviation list: ANC, Absolute neutrophil count; ALT, alanine aminotransferase; AST, aspartate aminotransferase; TG, triglyceride; HDL, high-density lipoprotein; BE, base excess;

Normal range: ANC > 1.5 × 10^9^/L; ALT,4-41U/L; AST,4-40U/L; TG,0.05–1.7 mmol/L; HDL 1.10–1.90 mmol/L; Uric acid 202.3–416.5 μmol/L; Fasting glucose 4.11–6.05 mmol/L; Lactic acid 0.50–2.20 mmol/L; Pyruvic acid 20–100 umol/L; PH 7.35–7.45; BE − 3 ~ 3

**Table 3:** Comparison of therapies and complications of 3 patients with GSD type Ib

	Patient 1			Patient 2			Patient 3		
	Clinical diagnosis	Genetic diagnosis	Last visit	Clinical diagnosis	Genetic diagnosis	Last visit	Clinical diagnosis	Genetic diagnosis	Last visit
Therapies									
Uncooked cornstarch	–	Irregular	Irregular	–	Regular	Regular	–	Regular	Regular
Neutropenia treatment	No	Yes	G-CSF	No	Yes	Yes	No	Yes	Yes
Sodium Bicarbonate Tablets	No	Yes	Yes	No	Yes	No	No	No	No
Complications									
Number of hospitalizations (times/year)	1	0.36	3	1	0	0	1	0	0
Inflammatory bowel disease	No	No	Yes	No	No	No	No	No	No
Mouth ulcers	Yes	Yes	Yes	Usually	Occasionally	Decrease	Occasionally	No	No
Upper respiratory tract infection	Yes	Yes	Yes	Yes	Less	Less	Repeatedly	Occasionally	Decrease
Pancreatitis	No	No	Yes	No	No	No	No	No	No
Other	–	–	Chronic superficial gastritis	–	–	–	–	–	–

### Patient 1

This female patient was admitted to hospital because of “abdominal distension for over 1 year” at the age of 15 months. At admission, her height SDS was − 2.26 and weight was 9 kg (10th–25th centile). There were abnormalities in absolute neutrophil count (ANC 1.07 × 10^9/L), liver function (ALT 75U/L, AST 103U/L), blood lipid (TG 8.84 mmol/L), fasting glucose (3.7 mmol/L), lactic acid (8.78 mmol/L), and blood gases (BE − 8.5). The liver ultrasound suggested hepatomegaly. She underwent liver biopsy and showed glycogen storage as confirmed by periodic acid-Schiff staining (PAS). She was also tested for glucose response to epinephrine stimulation after overnight fasting and the result was positive. Combined with the above results, she was clinically diagnosed as GSD. She was given oral uncooked cornstarch four daily doses of 1.0–2.0 g/kg (3am–9am–3pm–9pm). According to current guidelines, follow-up is recommended every 3–6 months (adjusted by disease changes and their ages). However, the patient did not take oral uncooked cornstarch regularly as prescribed. At the age of 12 years, she was finally diagnosed with GSD type 1b through genetic testing. She had an infection frequency of about 0.36 times/year before the diagnosis of genetic classification and was hospitalized for “repeated vomiting and diarrhea” 3 times in the past year, and had secondary “inflammatory bowel disease (IBD) and pancreatitis” during the last hospitalization (15 years old, course of disease 13.75 years). She received G-CSF treatment irregularly. At the last visit, the liver enzymes gradually returned to normal, triglycerides and lactic acid were decreased than before, but did not fall to normal levels. Her pyruvic acid and uric acid increased, and fasting hypoglycemia and metabolic acidosis have not been significantly improved.

### Patient 2

At the age of 3 years and a half, this male patient was admitted to hospital because of “hepatomegaly for over 3 years, upper respiratory tract infection for 1 week”. At admission, his height SDS was − 2.97 and weight was 13 kg (10th–25th centile). There were abnormalities in ANC 0.95 × 10^9 /L, liver function (ALT 50 U/L, AST 46 U/L), TG 5.53 mmol/L, fasting glucose (3.8 mmol/L), lactic acid (10.75 mmol/L), and blood gases (BE − 10.9). The liver ultrasound also suggested hepatomegaly. He also underwent liver biopsy and showed glycogen storage. He was tested for glucose response to epinephrine stimulation after overnight fasting and the result was positive, too. According to all the results, he was clinically diagnosed as GSD. He took oral uncooked cornstarch regularly. At 13 years old, he was diagnosed with GSD type 1b through genetic testing. At the last visit, the liver enzymes gradually returned to normal, triglycerides, lactic acid, pyruvic acid, and uric acid were decreased than before, but did not fall to normal levels. His fasting hypoglycemia and metabolic acidosis have been significantly improved. He had a history of oral ulcers. As the treatment time extended, the number of infections was significantly reduced, only presented with minor infections, such as upper respiratory tract infection or oral ulcers that did not require hospitalization.

### Patient 3

This female patient was admitted to hospital because of “short stature, hepatomegaly for 6 months” at the age of 3 years old. At admission, her height SDS was − 1.48 and weight was 15 kg (50th–75th centile). There were abnormalities in ANC (0.82 × 10^9 /L), TG (3.92 mmol/L), fasting glucose (3.48 mmol/L), lactic acid (9.61 mmol/L), and uric acid (519 μmol/L). The liver ultrasound suggested hepatomegaly. She also underwent liver biopsy and was tested for glucose response to epinephrine stimulation after overnight fasting and the results were positive. Therefore, she was clinically diagnosed as GSD. She was given oral uncooked cornstarch regularly. At the age of 10 years and a half, she was finally diagnosed with GSD type 1b through genetic testing. At the last visit, her triglycerides, lactic acid, pyruvic acid and uric acid were decreased than before. Her fasting hypoglycemia and metabolic acidosis have been significantly improved. She had a history of upper respiratory tract infections, about once a year.

### Genetic analysis

After obtaining the written informed consents, gene sequencing (Beijing MyGenostics Inc.) was performed on the probands and their parents. Our genetic testing strategy is a GSD panel based on target gene capture technology [6], 20 GSD genes reported in OMIM database (*GYS1, GYS2, G6PC, SLC37A4, GAA, AGL, GBE1, PYGM, PYGL, PFKM, PHKA2, PHKB, PHKG2, PHKA1, PGAM2, LDHA, ALDOA, ENO3, PGM1, GYG1, PRKAG2*) was used for GSDs. We fragmented the genomic DNA which extracted from the sample, and the DNA probes were designed to tile along the exon regions and exon–intron boundaries of the target genes. After enrichment of DNA fragments, Illumina HiSeq X ten sequencer was used for high-throughput sequencing of the captured exon region. Sanger sequencing was finally used to verify co-segregation in the family. Suspected candidate variants were screened by comprehensively considering the genetic pattern and the clinical characteristics of the disease. The pathogenicity of variants was predicted according to the 2015-ACMG Standards and Guidelines.

**Fig. 1:**
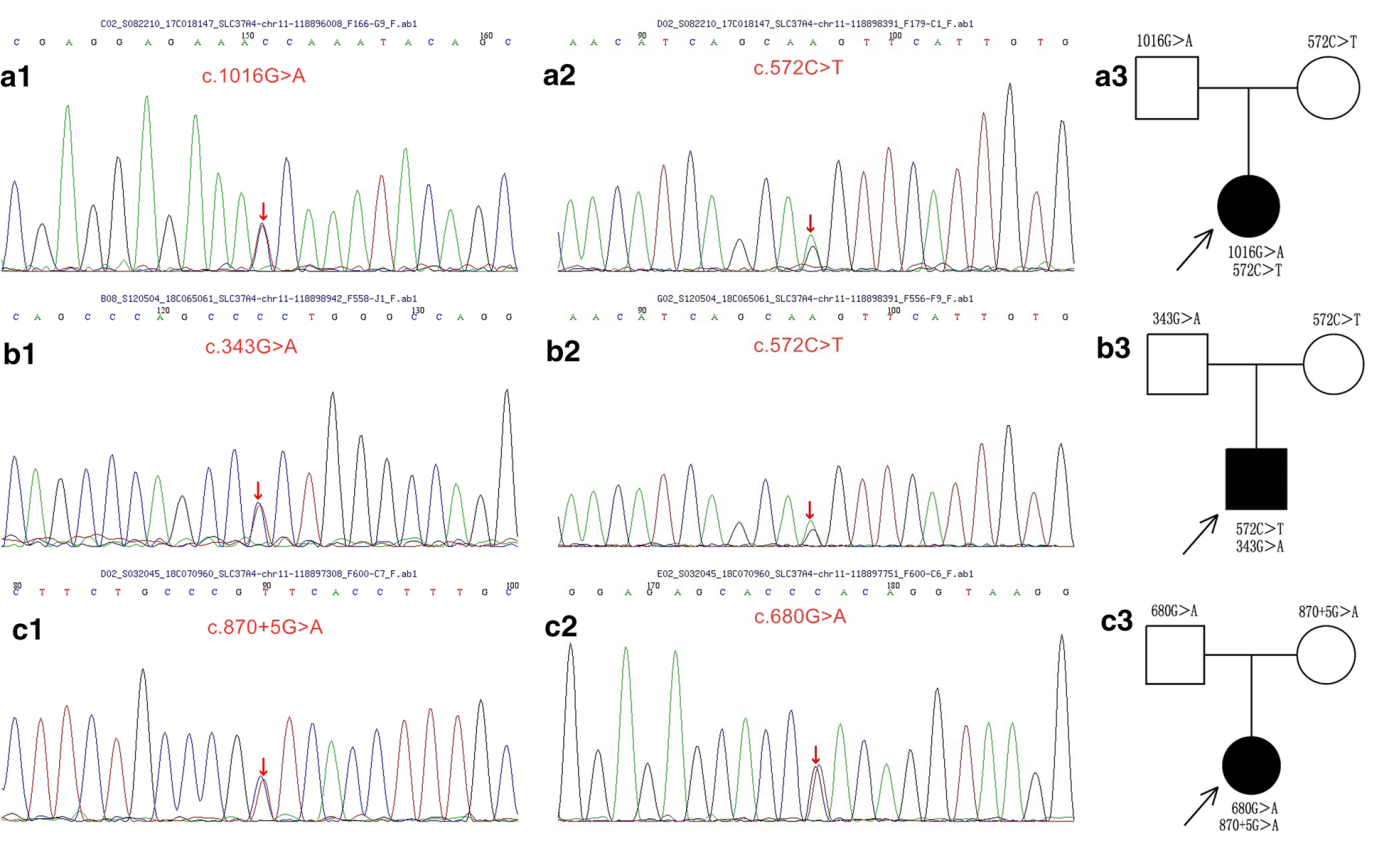
The exome sequencing results of the 3 patients and their parents. A1, patient 1 exon 10 sequencing showed variant c. 1016G > A (p.Gly339Asp) A2, patient 1 exon 6 sequencing showed variant c. 572C > T (p.Pro191Leu); B1, patient 2 exon 10 sequencing showed variant c. 343G > A (p.Gly115Arg), B2, patient 2 exon 6 sequencing showed variant c. 572C > T (p.Pro191Leu); C1, patient 3 exon 7 sequencing showed variant c. 680G > A (p.Trp227Ter), C2, patient 3 exon 7 sequencing showed variant c. 870 + 5G > A; A3, B3, and C3 are the pedigrees of the 3 patients

Five variants of *SLC37A4* gene were detected in 3 patients (see Table 1), including 3 missense variants, 1 frameshift variant, and 1 splicing variant, which were c.1016G > A (p.Gly339Asp), c.572C > T (p.Pro191Leu), c.343G > A (p.Gly115Arg), c.680G > A (p.Trp227Ter) and c.870 + 5G > A, respectively. Among them, c.680G > A was a novel variant. According to the ACMG guidelines, all the above gene variants were suspected disease-associated variants. Among them, the c.572C > T variant involved 2 patients (2/3) (see Table 1 and Fig. 1).

## Discussion and conclusions

Glycogen storage disease (GSD) Type Ib is a group of inherited metabolic disorders caused by variants in the *SLC37A4* gene, with an incidence of approximately 1/500 000. Fewer than 250 cases of type Ib patients have been reported, much less than type Ia, with the most reports in China, Japan, South Korea, Iran and Serbia [7, 8].

The human *SLC37A4* gene is located on chromosome 11q23, consists of 9 exons, spans approximately 5.3 kb of genomic DNA, and is expressed ubiquitously in liver, kidney, intestine, blood and skeletal muscle [9]. *SLC37A4* gene encodes G6PT, G6PT transports G6P from the cytoplasm to the lumen of the endoplasmic reticulum and delivers it to the catalytic site of G6Pase-α or G6Pase-β. G6Pase-α and G6Pase-β are G6P hydrolases in the endoplasmic reticulum membrane, which in turn hydrolyze G6P to glucose and inorganic phosphate [10]. Among them, G6Pase‑α and G6PT are coupled functionally, rather than physically, to maintain the interprandial (between meals) glucose homeostasis. A detrimental variant in the *SLC37A4* gene can cause G6PT deficiency or dysfunction, failing to complete the transport of G6P and resulting in disturbed glucose homeostasis, and then leading to hyperlipidemia, lactic acidosis, hyperuricemia, and other metabolic abnormalities. Whereas G6Pase-β couples functionally with G6PT to maintain neutrophil function and homeostasis. Consequently, G6PT is essential to maintain both interprandial glucose homeostasis and myeloid cell energy homeostasis [11]. Although G6Pase-α and G6Pase-β are similar in structure and function, patients with G6Pase-β deficiency do not exhibit the metabolic phenotypes of GSD I patients. In contrast, these individuals only present with severe congenital neutropenia syndrome, reflecting the differences between the presentations of GSD Ia and GSD Ib.

So far, there are 115 pathogenic variants in the *SLC37A4* gene that have been identified, including missense variants, nonsense variants, frameshift variants, splice site variants, and deletion variants, etc. There are ethnic variability in variant types and proportions [8, 12]. Previous studies have demonstrated that one of the most common types of variants is c.1042_1043del (p.Leu348Valfs* 53), which has been repeatedly reported in Germans (32%) and mixed Caucasians (27–31%) [2]. In the Korean population, the most common variant is c.443C > T (p.Ala148Val), which is found in 55.6% of GSD Ib patients and 38.9% of alleles. Since it has not been reported in other races, the author speculates that the variant may be unique to Koreans [12]. The common variant type in Japanese is c.352 T > C (p.Trp118Arg), accounting for 37–50% [12]. No strict genotype–phenotype correlation has been determined in previous studies [13–16]. In our study, 5 variant types of *SLC37A4* gene were detected in 3 patients. Among them, c.680G > A is a novel variant, the c.572C > T (p.Pro191Leu) variant is the most common and only reported in the Chinese population [17–19]. We speculate that c.572C > T may be a Chinese ethnicity -specific variant.

The typical clinical manifestations of GSD Ib are similar to those of type Ia, including impaired glucose homeostasis such as liver enlargement and growth retardation. By contrast, neutropenia and neutrophil dysfunction are major clinical phenotypes of patients with GSD Ib. GSD Ib patients are often accompanied by neutropenia and are prone to frequent infectious diseases, such as recurrent upper respiratory tract infections, oral ulcers, enterocolitis and inflammatory bowel disease (IBD). There are also reports of GSD Ib combined with Crohn's disease [20]. The exact mechanism of recurrent infections and IBD due to neutropenia and neutrophil dysfunction is still unclear. Studies have demonstrated that it may be related to impaired functions such as cell chemotaxis, calcium mobilization, respiratory burst, and leukocyte phagocytosis [21]. In addition, studies have shown that patients with GSD Ib are at increased risk of autoimmune diseases (including IBD, thyroid autoimmune diseases and myasthenia gravis, etc.). Melis et al. found that this may be related to a reduced engagement in T cell glycolysis and an impaired regulatory T cell function [22]. Therefore, GSD Ib caused by *SLC37A4* gene variants is both a metabolic and an immune disorder [1].

A decreased number of neutrophils in peripheral blood is an important feature that distinguishes GSD Ib from GSD Ia. It is worth mentioning that not all patients diagnosed with GSD Ib based on metabolic phenotypes and genetic testing develop neutropenia, which may be related to the residual transport activity of G6PT [1]. According to reports from different regions, the prevalence of neutropenia in GSD Ib patients is above 94%, and some patients may develop periodic neutropenia [1, 10, 12, 13]. There are scattered reports of atypical GSD Ib patients without neutropenia or infectious diseases [23, 24]. Neutropenia may also be observed in a subset of GSD Ia patients [25]. Therefore, it is not possible to distinguish between type Ib and Ia based on the decrease in the number of neutrophils alone.

The current treatment of GSD Ib is mainly symptomatic. As a serious metabolic and immune multisystem disorder, if not actively treated, it may cause the patient to be fatal in adolescence. Clinically, diet therapy (raw cornstarch, etc.) can maintain the patient's glucose stability and reduce the early symptoms of the disease [10, 21]. Those with poor diet control compliance often have obvious abnormalities in metabolic indicators, and death is mainly caused by metabolic disorders. Granulocyte colony stimulating factor (G-CSF) can improve neutropenia and IBD, but the underlying pathological process of the disease has not been corrected, and the specific mechanism is unknown [1, 2]. For type Ib patients with both IBD and neutropenia, G-CSF and 5-aminosalicylic acid can be used in combination [1]. In addition, GSD Ib patients receiving G-CSF treatment may have side effects such as splenomegaly, which is dose-dependent, and a few patients have myelodysplastic/acute myeloid leukemia [21, 26]. There is also a case report of severe hypertriglyceridemia (triglyceride 80 mmol/L) in a GSD Ib infant with a significant decrease in blood lipid levels after plasma exchange [15]. Another way to correct metabolic abnormalities in GSDI patients is liver transplantation or combined liver/kidney transplantation, while correction of bone marrow dysfunction in patients with GSD Ib can be achieved by bone marrow transplantation. However, many researchers believe that liver transplantation is a last resort, because the death rate associated with transplantation is higher than most other medical treatments [1]. Studies have reported that bone marrow transplantation for GSD Ib patients with severe IBD and repeated infections, although their neutropenia persists, neutrophil function and IBD are improved [27]. Although this is a case report, it offers hope for GSD Ib patients with severe myeloid complications. Since protein replacement therapy is not suitable for hydrophobic transmembrane proteins (such as G6PT), somatic gene therapy is a promising treatment for patients with type Ib. Effective use of gene therapy is very promising for correcting the metabolic abnormalities in GSD Ib patients, but to solve the problems of metabolic abnormalities and bone marrow complications at the same time, it may be necessary to construct either a vector with a wider range of tissue transduction specificity or a multivector approach [21].

The 3 patients in our study all had typical clinical manifestations such as elevated liver enzymes, fasting hypoglycemia, hyperlipidemia, hyperlactacidemia, lactic acidosis, and decreased neutrophil count, accompanied by hepatomegaly, growth retardation, and repeated infections. With increasing age, ANC of the 3 patients decreased progressively. Among them, the condition of patient 1 was poorly controlled, G-CSF was used irregularly, and the effect was not good, with secondary IBD and frequent hospitalizations due to infection. The other 2 patients were effectively controlled with age, and the number of infections was significantly decreased. Therefore, the clinical manifestations of patients with GSD Ib have certain heterogeneity.

In summary, GSD Ib patients have various gene variant types and different clinical symptoms. We reported 3 cases with GSD Ib and a novel *SLC37A4* variant. When recurrent upper respiratory tract infections or digestive tract symptoms are accompanied by hypoglycemia, dyslipidemia, metabolic disorders, elevated liver enzymes and/or neutropenia clinically, the possibility of GSD Ib should be vigilant.

## Data Availability

The *SLC37A4* variant can be found in NCBI Nucleotide under the accession number NM_001164277.2. The raw datasets generated and analysed during the current study are not publicly available in order to protect participant confidentiality. The datasets obtained during the current study are available from the corresponding author if the requirements are reasonable.

## Abbreviations

ALT: Alanine aminotransferase
AST: Aspartate aminotransferase
HDL cholesterol: High-density lipoprotein cholesterol
LDL cholesterol: Low-density lipoprotein cholesterol
TC: Total cholesterol
TG: Triglyceride

## References

[1] Chou JY, Jun HS, Mansfield BC (2015). Type I glycogen storage diseases: disorders of the glucose-6-phosphatase/glucose-6-phosphate transporter complexes. J Inherit Metab Dis.

[2] Kishnani PS, Austin SL, Abdenur JE, Arn P, Bali DS, Boney A (2014). Diagnosis and management of glycogen storage disease type I: a practice guideline of the American College of Medical Genetics and Genomics. Genet Med.

[3] Ihara K, Nomura A, Hikino S, Takada H, Hara T (2000). Quantitative analysis of glucose-6-phosphate translocase gene expression in various human tissues and haematopoietic progenitor cells. J Inherit Metab Dis.

[4] Jun HS, Lee YM, Cheung YY, McDermott DH, Murphy PM, De Ravin SS (2010). Lack of glucose recycling between endoplasmic reticulum and cytoplasm underlies cellular dysfunction in glucose-6-phosphatase-beta-deficient neutrophils in a congenital neutropenia syndrome. Blood.

[5] Visser G, Rake JP, Labrune P, Leonard JV, Moses S, Ullrich K (2002). Consensus guidelines for management of glycogen storage disease type 1b—European Study on Glycogen Storage Disease Type 1. Eur J Pediatr.

[6] Liang Y, Du C, Wei H, Zhang C, Zhang M, Hu M, et al. Genotypic and clinical analysis of 49 Chinese children with hepatic glycogen storage diseases. Mol Genet Genomic Med. 2020;8:e1444. 10.1002/mgg3.1444 PMC7549605 32772503

[7] Bali DS, Chen Y-T, Austin S, Goldstein JL. Glycogen Storage Disease Type I. In: Adam MP, Ardinger HH, Pagon RA, et al, eds GeneReviews® Seattle (WA): University of Washington, Seattle. 2006 April 19.

[8] Skakic A, Djordjevic M, Sarajlija A, Klaassen K, Tosic N, Kecman B (2018). Genetic characterization of GSD I in Serbian population revealed unexpectedly high incidence of GSD Ib and 3 novel SLC37A4 variants. Clin Genet.

[9] Annabi B, Hiraiwa H, Mansfield BC, Lei KJ, Ubagai T, Polymeropoulos MH (1998). The gene for glycogen-storage disease type 1b maps to chromosome 11q23. Am J Hum Genet.

[10] Chou JY, Jun HS, Mansfield BC (2010). Glycogen storage disease type I and G6Pase-beta deficiency: etiology and therapy. Nat Rev Endocrinol.

[11] Yuan Y, Liu Y, Qiu Z (2017). Analysis of SLC37A4 gene in 3 cases of glycogen storage disease type Ib. J Clin Pediatr.

[12] Choi R, Park HD, Ko JM, Lee J, Lee DH, Hong SJ (2017). Novel SLC37A4 mutations in korean patients with glycogen storage disease Ib. Ann Lab Med.

[13] Melis D, Fulceri R, Parenti G, Marcolongo P, Gatti R, Parini R (2005). Genotype/phenotype correlation in glycogen storage disease type 1b: a multicentre study and review of the literature. Eur J Pediatr.

[14] Sarajlija A, Djordjevic M, Kecman B, Skakic A, Pavlovic S, Pasic S (2020). Impact of genotype on neutropenia in a large cohort of Serbian patients with glycogen storage disease type Ib. Eur J Med Genet.

[15] You C, Fu Y (2019). GUlycogen storage disease type I b with severe hypertriglyceridemia due to SLC37A4 gene mutation: a case report and literature review. Chin J Evid Based Pediatr.

[16] Qiu Z, Lu C, Wang W, Qiu J, Wei M (2011). Mutation in the SLC37A4 gene of glycogen storage disease type Ib in 15 families of the mainland of China. Chin J Pediatr.

[17] Lam CW, Chan KY, Tong SF, Chan BY, Chan YT, Chan YW (2000). A novel missense mutation (P191L) in the glucose-6-phosphate translocase gene identified in a Chinese family with glycogen storage disease 1b. Hum Mutat.

[18] Yuen Y-P, Cheng W-F, Tong S-F, Chan Y-T, Chan Y-W, Lam C-W (2002). Novel missense mutation (Y24H) in the G6PT1 gene causing glycogen storage disease type 1b. Mol Genet Metab.

[19] Tong W, Wang Y, Lu Y, Ye T, Song C, Xu Y (2018). Whole-exome Sequencing Helps the Diagnosis and Treatment in Children with Neurodevelopmental Delay Accompanied Unexplained Dyspnea. Sci Rep.

[20] Xu X, Xiao Y, Qiu W, Guo Y, Wang X, Xu C (2017). A case of glycogen storage disease type Ib complicated with Crohn's disease. Chin J Pediatr.

[21] Chou JY, Cho JH, Kim GY, Mansfield BC (2018). Molecular biology and gene therapy for glycogen storage disease type Ib. J Inherit Metab Dis.

[22] Melis D, Carbone F, Minopoli G, La Rocca C, Perna F, De Rosa V (2017). Cutting edge: increased autoimmunity risk in glycogen storage disease Type 1b is associated with a reduced engagement of glycolysis in T cells and an impaired regulatory T cell function. J Immunol.

[23] Kure S, Hou DC, Suzuki Y, Yamagishi A, Hiratsuka M, Fukuda T (2000). Glycogen storage disease type Ib without neutropenia. J Pediatr.

[24] Angaroni CJ, Labrune P, Petit F, Sastre D, Capra AE, Dodelson de Kremer R, et al. Glycogen storage disease type Ib without neutropenia generated by a novel splice-site mutation in the glucose-6-phosphate translocase gene. Mol Genet Metab. 2006;88(1):96–9. 10.1016/j.ymgme.2005.12.011 16490377

[25] Weston BW, Lin JL, Muenzer J, Cameron HS, Arnold RR, Seydewitz HH (2000). Glucose-6-phosphatase mutation G188R confers an atypical glycogen storage disease type 1b phenotype. Pediatr Res.

[26] Khalaf D, Bell H, Dale D, Gupta V, Faghfoury H, Morel CF (2019). A case of secondary acute myeloid leukemia on a background of glycogen storage disease with chronic neutropenia treated with granulocyte colony stimulating factor. JIMD Rep.

[27] Pierre G, Chakupurakal G, McKiernan P, Hendriksz C, Lawson S, Chakrapani A (2008). Bone marrow transplantation in glycogen storage disease type 1b. J Pediatr.

